# Newcastle Disease Virus at the Forefront of Cancer Immunotherapy

**DOI:** 10.3390/cancers12123552

**Published:** 2020-11-28

**Authors:** Bharat Burman, Giulio Pesci, Dmitriy Zamarin

**Affiliations:** 1 Department of Medicine, Gynecologic Medical Oncology Service, Memorial Sloan Kettering Cancer Center, New York, NY 10065, USA; burmanb@mskcc.org (B.B.); pescig@mskcc.org (G.P.); 2 Ludwig Collaborative Laboratory, Memorial Sloan Kettering Cancer Center, New York, NY 10065, USA; 3 Department of Medicine, Weill-Cornell Medical College, New York, NY 10065, USA; 4 Parker Institute for Cancer Immunotherapy, Memorial Sloan Kettering Cancer Center, New York, NY 10065, USA

**Keywords:** oncolytic virus, newcastle disease virus, NDV, cancer, immunotherapy, immune checkpoint inhibitor, PD-1, PD-L1, CTLA-4, type I interferon

## Abstract

**Simple Summary:**

Newcastle disease virus (NDV) is an RNA virus belonging to the Paramyxoviridae family. In nature, NDV primarily infects birds, but poses no threat to human health. Multiple studies have demonstrated that NDV caries oncolytic potential due to its predilection for infection and replication in human cancer cells while sparing normal cells. In addition to its direct lytic effects, the virus triggers both innate and adaptive immune responses. In animal models, NDV injection into a tumor has been demonstrated to result in local inflammation and the recruitment of tumor-specific T cells, an effect that can be further potentiated through the use of viruses encoding immunomodulatory ligands and through combinations with immune checkpoint blockade. Initial clinical trials with naturally occurring NDV administered intravenously demonstrated durable responses across a number of cancer types. Clinical studies utilizing recombinant NDV in combination with immune checkpoint inhibitors are ongoing.

**Abstract:**

Preclinical and clinical studies dating back to the 1950s have demonstrated that Newcastle disease virus (NDV) has oncolytic properties and can potently stimulate antitumor immune responses. NDV selectively infects, replicates within, and lyses cancer cells by exploiting defective antiviral defenses in cancer cells. Inflammation within the tumor microenvironment in response to NDV leads to the recruitment of innate and adaptive immune effector cells, presentation of tumor antigens, and induction of immune checkpoints. In animal models, intratumoral injection of NDV results in T cell infiltration of both local and distant non-injected tumors, demonstrating the potential of NDV to activate systemic adaptive antitumor immunity. The combination of intratumoral NDV with systemic immune checkpoint blockade leads to regression of both injected and distant tumors, an effect further potentiated by introduction of immunomodulatory transgenes into the viral genome. Clinical trials with naturally occurring NDV administered intravenously demonstrated durable responses across numerous cancer types. Based on these studies, further exploration of NDV is warranted, and clinical studies using recombinant NDV in combination with immune checkpoint blockade have been initiated.

## 1. Introduction

Observations that naturally occurring viral infections could cause spontaneous tumor regressions led to the search for viruses that could selectively lyse tumor cells with limited pathogenicity in humans [1]. In the 1950s, it was discovered that Newcastle disease virus (NDV), a highly virulent pathogen to over 240 species of birds, has oncolytic properties [2,3]. A decade later, NDV was injected intraperitoneally in mice with Ehrlich ascites, leading to tumor cell lysis and durable immunity upon tumor re-challenge [4,5]. Around the same time, NDV was tested clinically in a patient with acute myelogenous leukemia, who experienced transient anti-leukemic effect and clinical improvement with limited side effects [6].

NDV is an avian paramyxovirus type I virus belonging to the *Avulavirus* genus. NDV has a spherical morphology, formed by a lipid bilayer which surrounds the RNA genome. The genome consists of a 15,186-nucleotide negative single-strand RNA encoding six different genes: nucleocapsid protein (NP), phosphoprotein (P), matrix protein (M), fusion protein (F), haemagglutinin-neuraminidase (HN), and RNA-dependent RNA polymerase (L). NP, P, and L proteins form a ribonucleotide protein complex that embeds the genomic RNA. The lipid envelope surrounds the ribonucleotide protein complex [7,8,9]. NDV infection is initiated by binding of the viral surface HN and F glycoproteins to sialic acid-containing host cell surface proteins [10,11]. This triggers a conformational change in the F protein, which results in fusion of the viral envelope and the cell plasma membrane. Viral particles are internalized by endocytosis, and adjacent cells with attached particles may form syncytia due to the fusogenic F protein [8,11,12]. After viral entry, the M protein dissociates from the ribonucleotide protein complex in the cytoplasm, and the P and L proteins form a polymerase complex that initiates transcription of the viral RNA [10,13].

There are three main pathotypes of NDV, classified by the severity of disease caused in birds: lentogenic (avirulent), mesogenic (intermediate), and velogenic (highly virulent) [8]. Virulence is primarily determined by sequence variation in the F gene, which affects F protein cleavage efficiency [14,15]. Lentogenic viruses possess a monobasic F cleavage site and exhibit reduced capacity for multicycle replication and lysis. The mesogenic and velogenic NDV types possess a polybasic F cleavage site and have superior capacity for multicycle replication, syncytia formation, and tumor cell lysis. In birds, mesogenic strains cause mild respiratory and gastrointestinal disease, while velogenic strains cause severe respiratory and gastrointestinal disease as well as neurotoxicity [14,15,16]. In preclinical studies, the most commonly used strains are the mesogenic strains MTH-68/H, PV701, 73T, Italien, Beaudette C, and AF2240, and the lentogenic strains HUJ, Ulster, LaSota, Hitchner B1, and V40-UPM. Among these strains, the lentogenic NDV LaSota strain is a proven and safe vaccine vector that is commonly used as a live attenuated vaccine in the poultry industry [17]. Due to capacity for multicycle replication, mesogenic and velogenic exhibit superior capacity for direct virus-mediated lysis. It is incorrect, however, to classify the lentogenic NDV strains as completely nonlytic. In a number of studies using lentogenic NDV strains lacking the polybasic F cleavage site, the viruses still demonstrate capacity to infect and lyse cancer cells at multiplicity of infection as low as 0.001 [18].

Oncolytic properties of NDV derive primarily from deficient type I IFN signaling pathways and less sensitive type I IFN receptor-mediated signaling in tumor cells [19,20,21]. Mutations in genes related to the type I IFN pathway and the downstream Janus kinase (JAK)/signal transducer and activator of transcription (STAT) pathway are associated with NDV susceptibility and cytotoxicity [19,22,23]. Tumor cell susceptibility to NDV infection may also be based on the presence of sialic acid-containing cell surface proteins. It was proposed that the combination of altered type I IFN-related gene expression and sialic acid content could act as a clinical biomarker for determining susceptible tumor types [24]. Finally, defects in apoptotic pathways such as the Fas-FasL interaction or overexpression of antiapoptotic genes such as Livin and BcL-xL, which are documented in many tumor types, may increase susceptibility to NDV allowing for viral persistence, increased replication, and spread to surrounding cells [25,26,27].

NDV has been shown to cause cell death by apoptosis, necrosis, or autophagy mechanisms [26,28,29,30]. Viral HN protein can directly trigger the release of type I IFN and upregulates tumor necrosis factor (TNF)-related apoptosis inducing ligand (TRAIL) [31]. In human peripheral blood mononuclear cells (PBMCs), TRAIL signaling in turn upregulates apoptotic genes (FasL, Bax, caspase-8, caspase-9, and caspase-3) [32]. HN gene expression alone has been reported to induce apoptosis in human breast cancer MCF-7 cells [33]. NDV can also induce apoptosis through interferon-independent mechanisms such as the intrinsic mitochondrial death pathway [34]. Finally, the formation of syncytia by some NDV strains (termed “fusogenic” strains) ultimately leads syncytium disintegration either through necrosis or apoptosis [35].

## 2. Activation of the Innate Anti-Tumor Immune Response by NDV

The type I IFN pathway plays a central role in mediating antiviral immunity in mammals [36]. Type I IFNs have antiviral, proapoptotic, and immunomodulatory effects, all of which contribute in large part to the mechanism by which NDV induces antitumor response [36,37]. Type I IFN production in response to viral infection within the tumor microenvironment may have direct antiproliferative effects in some tumors [38]. More significantly, type I IFN signaling activates both innate and adaptive immunity through recruitment of innate cells including natural killer (NK) cells and antigen-presenting cells (APCs), upregulation of cell adhesion, major histocompatibility complex (MHC) and costimulatory molecules, and priming of antigen-specific T cells [37,39,40,41,42]. Thus, activation of type I IFN signaling is one of the key pathways being explored for cancer immunotherapy, and this is supported by the findings that tumors with high CD8+ T cell proliferation and responsiveness to immune checkpoint inhibitors are enriched for genes associated with type I IFN signaling [43].

**Figure 1:**
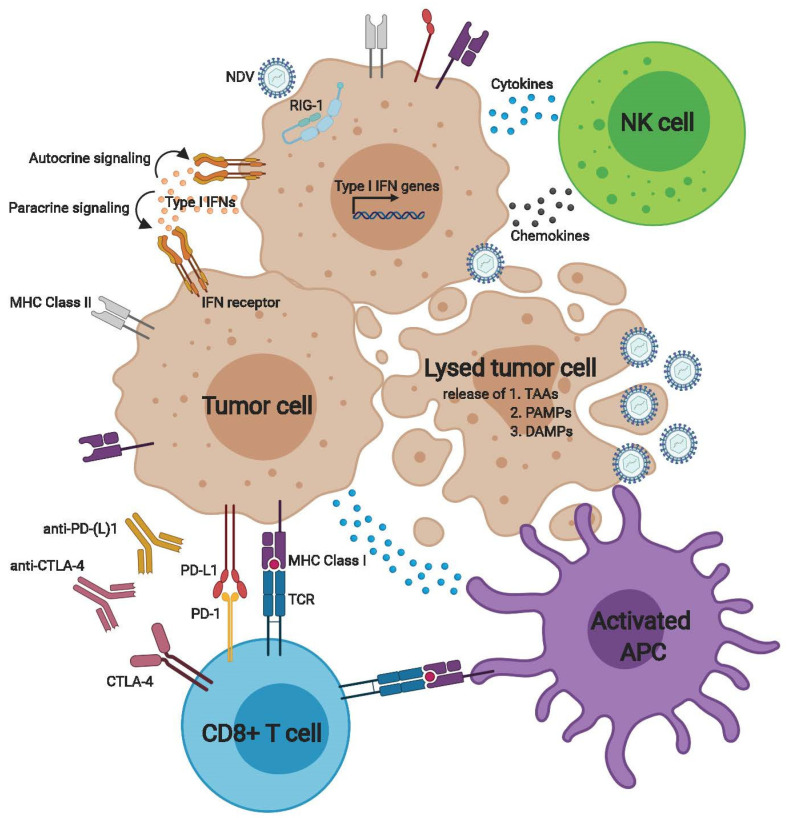
Newcastle disease virus (NDV) activates innate and adaptive anti-tumor immune responses. NDV selectively infects tumor cells that have defective anti-viral defenses. Extracellular and intracellular signaling mediated by sensors such as the RNA helicase RIG-1 leads to expression of type I IFN and related genes. Autocrine and paracrine IFN signaling upregulates MHC class I and II presentation, co-stimulatory molecules, and immune checkpoints on the cell surface. The release of cytokines and chemokines in addition results in the recruitment of innate effector cells such as NK cells and macrophages and antigen-presenting cells (APCs). Virus-mediated direct oncolysis leads to release of tumor antigens, PAMPs, and DAMPs that activate APCs including dendritic cells capable of antigen cross-presentation. Activated APCs prime T cells, resulting in generation of cytolytic T cells directed toward tumor and viral antigens; however, effector function of the activated T cells can be inhibited by upregulation of PD-L1 on tumor cells and APCs, and PD-1 and CTLA-4 on T cells. Upregulation of these negative feedback mechanisms provide the rationale for combining NDV with immune checkpoint inhibitors.

Upon NDV infection, pathogen-associated molecular patterns (PAMPs) inherent to the virus and danger-associated molecular patterns (DAMPs) released by dying cells are recognized by pattern recognition receptors (PRRs) including extracellular Toll-like receptors (TLRs) 3, 7, 8, and 9; intracellular nucleotide-binding oligomerization domain (NOD) proteins; and intracellular RNA helicases such as RIG-1 or MDA5 [44,45] (Figure 1). Recognition of PAMPs and DAMPs by PRRs leads to the activation of transcription factors including IFN regulatory factor (IRF)3, IRF7, and nuclear factor kappa B via the adaptors interferon β stimulator-1 and stimulator of interferon genes (STING) [44]. This signaling cascade results in the transcription and expression of genes encoding proinflammatory cytokines and type I and type III IFN proteins [19,44]. In the case of NDV, cytosolic RNA generated by NDV infection is sensed by RIG-1, and reduction of RIG-1 protein levels has been shown to correlate with decreased intensity of type I IFN response to NDV in vitro [23,46] (Figure 1).

Tumor cells often have impaired type I IFN signaling, which is one of the principal mechanisms resulting in increased tumor cell sensitivity to NDV infection. Despite these deficiencies, the impairment in type I IFN production is typically not absolute, especially as NDV is capable of infecting normal cells in the tumor microenvironment, which have preserved type I IFN response [46,47,48]. Transcriptional profiling of mouse tumors injected with NDV reveals upregulation of type I IFN response-related genes and a range of cytokines and chemokines that mediate recruitment and proliferation of innate and adaptive immune cells [47,49]. Interestingly, this signature was shown to be independent of NDV-mediated replicative or lytic potential in a study utilizing the lentogenic NDV LaSota strain, indicating that type I IFN signaling activated to even a limited virus infection is sufficient to drive the inflammatory response [49].

While a strong type I IFN response to NDV results in a proinflammatory tumor microenvironment that contributes to the antitumor response, it may, on the other hand, limit therapeutic efficacy by suppressing NDV replication and virus-mediated lysis. Indeed, pretreatment with type I IFN has been shown to limit NDV replication in some tumor cell lines [20,23,46,48]. Therefore, a key unanswered question in the field concerns the timing of type I IFN induction, whereby a balance should be achieved between adequate virus replication and tumor lysis and induction of innate immune response to promote further adaptive immunity. A recombinant lentogenic NDV strain (Hitchner B1) expressing the influenza A virus IFN antagonist protein NS1, which suppresses RIG-1 receptor signaling, IRF3 dimerization, and expression of IFN-β, potently reduced IFN signaling across a panel of cancer cell lines and resulted in increased NDV replication and cytolysis [50]. In vivo, this virus was more effective in controlling tumor growth and prolonging survival in a syngeneic melanoma mouse model [50]. Similar results were demonstrated using the recombinant mesogenic Beaudette C NDV strain expressing an IFN-antagonist protein which showed higher efficiency in tumor regression in a xenotransplanted fibrosarcoma mouse model [47]. Despite these findings, type I IFN has been shown to be essential for antitumor activity of NDV, and in mice lacking type I IFN receptor, the virus exhibited no ability to control tumor growth [51].

In addition to activation of tumor cell-inherent type I IFN signaling, the inflammatory environment generated by NDV results in the recruitment of innate effector cells and adaptive immune cells (discussed below) that contribute to antitumor immunity (Figure 1). In particular, intratumoral NDV injection leads to a significant tumor infiltration with natural killer (NK) cells [42,52,53]. Interestingly, depletion of NK cells prior to NDV treatment in a syngeneic mouse tumor model abrogated antitumor efficacy, while depletion of NK cells concomitantly with NDV treatment did not, suggesting that while NK cells are important early responders to NDV infection, their role appears to be essential only for the initial inflammatory response [53,54]. Last, NDV infection also results in the recruitment of myeloid cells, which have important roles in phagocytosis and antigen presentation [54,55].

## 3. Activation of the Adaptive Antitumor Immune Response by NDV

Activation of the innate immune system, largely mediated by type I IFN signaling in response to NDV infection, provides optimal conditions for stimulating adaptive antitumor immunity. Secretion of inflammatory mediators leads to the recruitment of both myeloid and lymphoid cells to the tumor microenvironment [41] (Figure 1). A key effector population is dendritic cells (DCs), a subset of which specialize in antigen cross-presentation (BATF3-dependent or CD8+ DCs) and priming of antigen-specific CD8+ T cells [43,56,57]. NDV infection can cause cell death by apoptosis, necrosis, or autophagy, all of which can lead to the release of viral and tumor-associated antigens and debris within the tumor microenvironment. Cross-presenting DCs become activated and mature in response to uptake of these antigens and in response to PAMPs and DAMPs [43,56,57]. Interleukin (IL)-12 produced by cross-presenting DCs, acting in concert with type I IFN signaling in the tumor microenvironment, leads to upregulation of MHC class I and II molecules, cell adhesion molecules, and co-stimulatory molecules, all of which promote priming of T cells by APCs [56,58]. In effect, tumor infection with NDV acts as an in situ vaccine by causing the release and presentation of tumor antigens in a setting of an inflammatory environment, eliminating the need for selection of antigens needed with other vaccine modalities [59] (Figure 1).

Evidence for NDV-induced tumor antigen-specific CD8+ T cell response comes from studies involving bilateral flank syngeneic tumor models, whereby lentogenic NDV LaSota strain is administered to a single flank tumor [49,51,53,54,60]. Due to restriction of virus replication to the injected tumor, such models allow for assessment of both local and distant immune effects. Interestingly, intratumoral therapy with NDV resulted in a marked increase in CD4+ and CD8+ T cell infiltration in both injected and non-injected tumors. Importantly, there was a greater increase in CD4+FoxP3− cells as compared to regulatory CD4+FoxP3+ cells [49,51,53,54,60]. Furthermore, tumor-infiltrating T cells isolated from both tumor sites expressed increased activation, proliferation, and lytic markers [51,54,60]. This was further supported by the finding of increased expression of other genes associated with T cell activation within the tumor microenvironment of both the injected and non-injected lesions [60]. Importantly, this expression profile was not observed in the spleen, suggesting that the activated T cell response was specific to tumors and not due to nonspecific inflammation [54]. Last, intratumoral NDV therapy resulted in tumor growth delay of both virus-injected and distant tumors and prolonged animal survival, implicating potential development of systemic tumor antigen-specific T cell responses [51]. Overall, these findings are consistent with clinical observations of intralesional administration of talimogene laherparepvec (T-VEC) in advanced melanoma leading to tumor immune infiltration and regression of both injected lesions and distant sites [61].

In the experiments discussed above, complete tumor regressions in the contralateral non-injected tumors were rare despite a marked increase in T cell infiltration, suggesting that compensatory immune inhibitory mechanisms may dampen the immune response. Indeed, upregulation of a number of immune checkpoints, including CTLA-4 and PD-1, was observed on tumor-infiltrating T cells in both virus-injected and distant tumors [51,54]. In addition, upregulation of PD-L1 was observed on tumor, myeloid, and stromal cells [54]. PD-L1 increase occurred early in the injected tumor and was found to be due to rapid upregulation of type I IFN in response to NDV injection. High levels of PD-L1 were also found in the distant non-injected lesion, albeit later in the treatment course, and were found to be upregulated in response to increase in tumor infiltrating lymphocytes. Interestingly, PD-L1 expression in distant tumors was more common in myeloid cells than in tumor cells [54]. Overall, these findings highlighted the rationale for combining NDV with immune checkpoint inhibitors as a means to alleviate the negative feedback mechanisms likely impacting therapeutic efficacy [41]. Indeed, combination of NDV with systemic anti-CTLA-4, anti-PD-1, or anti-PD-L1 resulted in enhanced rejection of bilateral tumors and prolonged animal survival compared to either treatment alone, an effect that was seen in multiple tumor types [51,54]. These findings highlight that intratumoral therapy with NDV can be an effective strategy to drive systemic efficacy of immune checkpoint inhibitors and have now been confirmed across a number of oncolytic viruses [61,62,63,64,65,66,67,68,69,70,71,72,73], including early clinical studies of immune checkpoint inhibitors in combination with T-VEC [61,74,75].

Despite these findings, the responses to oncolytic viruses in clinical trials have not been universal, and our understanding of the mechanisms by which oncolytic viruses activate antitumor immunity remains limited. For example, replicative capacity of oncolytic viruses is a subject of ongoing debate in the oncolytic virus field. As well-replicating viruses tend to exhibit superior lytic ability, many groups prefer well-replicating oncolytic viruses as a means to achieve a maximal tumor-debulking effect through direct virus-mediated lysis [76]. However, it is unclear how replicative capacity alters antitumor immunity. In human bladder cancer cell lines infected with lentogenic NDV LaSota strain, upregulation of innate immune response and antigen presentation machinery was not related to virus replication or tumor lysis [49]. Furthermore, intratumoral NDV therapy in the MB49 bladder cancer model, which is poorly susceptible to NDV-mediated lysis, resulted in complete regression of both virus-injected and distant tumors when used in combination with immune checkpoint inhibitors [49].

Related to the question of replicative capacity is the question of the impact of pre-existing anti-viral immunity. Adaptive immune responses towards an oncolytic virus can curtail anti-tumor efficacy by limiting virus persistence, replication and lysis [77,78]. While immunization of mice with NDV LaSota led to the development of neutralizing antibodies resulting in decreased NDV replication with subsequent challenge, antitumor efficacy was not compromised and, on the contrary, was superior in pre-immunized mice [53]. This was supported by increased T cell infiltration including T-helper cells and upregulation of immune-related gene expression in both treated and distant tumors [53]. Several potential mechanisms could contribute to enhanced antitumor efficacy observed with pre-existing immunity, including an antiviral memory response resulting in more rapid induction of tumor inflammatory response, bystander killing from virus-directed T cells, and epitope spreading [53,71,79]. A closer examination of antitumor versus antiviral immune responses elicited by NDV will be needed to answer these questions. In addition, further studies will be needed to understand if pre-existing antiviral immunity potentiates the antitumor response only within the setting of intratumoral therapy, although some patients who received systemically administered NDV in prior clinical trials experienced durable responses, the onset of which happened late in the treatment course [80,81].

## 4. Engineering NDV to Modulate Innate and Adaptive Immune Responses

**Figure 2:**
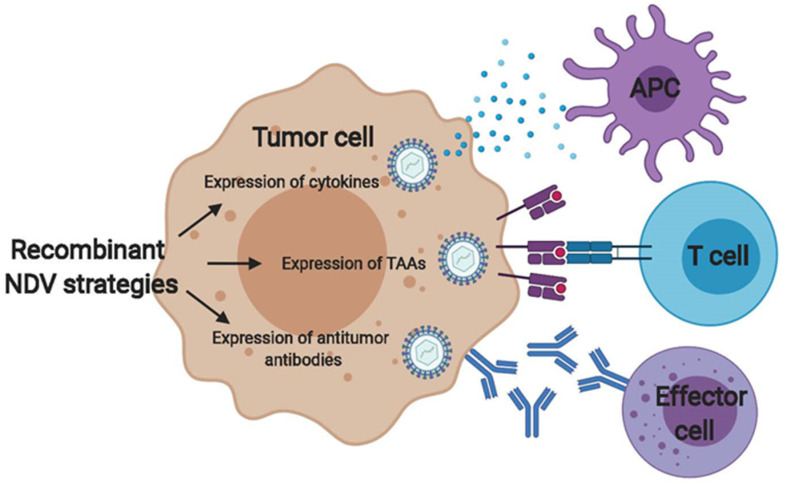
Strategies to enhance the NDV antitumor immune response by recombinant genetic engineering. Genetic engineering can be used to generate NDV strains with greater potential to stimulate antitumor immune response. First, NDV engineered to express cytokines such as GM-CSF or interleukins can increase recruitment of innate effector cells such as antigen-presenting cells (APCs). Second, NDV can be used as a therapeutic vaccine targeted to specific tumor antigens such as oncogenic viral antigens, frame shift mutations, or mutated self-antigens. Third, NDV can be engineered express single-chain variable fragments or full antitumor antibodies to induce antibody-dependent cellular cytotoxicity by effector cells.

With the development of reverse genetics, it has become possible to modify the NDV viral genome and introduce foreign sequences to potentially enhance oncolytic and immunostimulatory properties of these agents [82]. Several strategies to enhance innate and/or adaptive antitumor immunity by engineering NDV to express cytokines, antibodies, ligands, or tumor antigens have been explored, and a few are reviewed below (Figure 2). Given its ability to activate antigen-presenting cells, granulocyte–macrophage colony stimulating factor (GM-CSF) has been explored as a therapeutic transgene within the context of multiple oncolytic viruses, and T-VEC, an oncolytic herpes simplex virus expressing GM-CSF, was approved by the FDA for treatment of metastatic melanoma [74]. A recombinant strain based on the mesogenic NDV 73T strain currently in clinical development, MEDI5395, expressing human GM-CSF was recently shown to increase secretion of pro-inflammatory cytokines such as IFN-α, IL-6, IL-8, and TNF-α in PBMC samples from healthy volunteers, and stimulated PBMCs to exert antitumor effects in vitro [83]. In addition, infection of dendritic cells led to their maturation, and co-culture of dendritic cells with allogeneic T cells increased the levels of T cell effector cytokines IL-2 and IFN-γ [83]. In a separate study using NDV Hitchner B1 strains engineered to express either murine IL-2, IFN-γ, and GM-CSF in vivo, only NDV expressing IL-2 led to a significant increase in overall animal survival when compared to parental NDV [82]. Similar results were recently demonstrated with a lentogenic recombinant NDV strain expressing IL-24 [84].

Optimal immune mechanisms for intratumoral targeting with oncolytic virus are unknown. Gene expression profiling of tumors after NDV injection revealed the upregulation of T cell co-stimulatory receptors ICOS, 4-1BB, GITR, OX40, CD27, and CD40, all of which are currently being evaluated as therapeutic targets in clinic using monoclonal antibodies [60]. Targeting of ICOS within the context of tumor microenvironment using engineered cellular vaccines expressing ICOS ligand (ICOSL) has in particular been previously demonstrated to improve systemic efficacy of CTLA-4 blockade through potentiation of cytotoxic T cell function [85]. Intratumoral administration of engineered NDV LaSota expressing ICOSL resulted in enhanced infiltration of CD8+ and CD4+ T cells, tumor growth delay of both injected and non-injected tumors, and prolonged survival, as compared to wild type NDV, and this effect that was further enhanced when combined with anti-CTLA-4 blockade [60]. These findings highlight that stimulation of both innate and adaptive immune response pathways within the context of intratumoral NDV therapy may be required for optimal activation of antitumor immune response. Recently, recombinant NDV LaSota strains expressing soluble single-chain variable fragments for anti-CD28, anti-PD1, and anti-PDL1 were generated, as well as versions fused to IL-12 [86]. All of these strains showed improved tumor control and survival in a melanoma mouse model [86].

Engineering NDV to express a tumor-associated antigen represents another attractive strategy due to its potential to overcome immune tolerance within the context of NDV-induced inflammatory environment [59]. Such strategy was explored with NDV Hitchner B1 expressing an MHC class I restricted epitope of β-galactosidase (β-gal), a model antigen expressed by murine CT26 colorectal carcinoma cells [87]. Intratumoral therapy of CT26 tumor-bearing mice induced a β-gal-specific immune response and significant increase in the number of complete tumor regressions compared to parental NDV. This response was further boosted by co-administration of NDV expressing IL-2, with 90% tumor regression seen [87]. These findings warrant investigation of NDVs expressing other tumor-associated antigens, such as those caused by oncogenic viral antigens, frame shift mutations, and mutated self-antigens, but also highlight that combinatorial strategies using oncolytic viruses targeting different mechanisms (e.g., antigens and adaptive immunity) may be required to achieve optimal anti-tumor response.

## 5. Clinical Experience with NDV

The immunogenic properties of NDV were recognized early, and a number of studies have explored the virus for immunization of patients with virus-modified cancer cell vaccines [88,89,90,91,92,93,94,95,96,97,98,99,100,101,102,103,104,105,106]. Many of the early studies were performed by William Cassel and colleagues utilizing autologous or allogeneic NDV oncolysates for vaccination of patients with resected high risk melanoma, demonstrating improvement in overall survival when compared to historical controls [88,89,93,101,107]. A similar strategy was developed by Volker Schirrmacher and colleagues, where whole-cell autologous irradiated tumor cells were modified by infection with attenuated NDV [108]. The investigators evaluated vaccination with NDV-modified tumor cells in adjuvant or advanced disease setting across a number of cancers, demonstrating evidence of antitumor immunity (measured by delayed type hypersensitivity) and improvement in survival in some studies [91,92,94,95,98,109]. A similar approach was used by Liang and colleagues in a phase III trial in colorectal cancer, comparing adjuvant immunization with NDV-modified autologous cancer cells to resection alone [103]. The study reported improvement in overall survival in the vaccine group (7 vs. 4.5 years), which was statistically significant. Overall, these studies provide a proof of concept that infection of cancer cells by NDV can enhance cancer cell immunogenicity and has a potential to stimulate anti-tumor immunity. While the majority of the studies above are plagued by lack of control arms, prospective randomized studies are certainly warranted, especially in combination with modern immunotherapy agents such as immune checkpoint inhibitors.

As preparation of autologous virus-modified vaccines can be cumbersome, a number of studies have explored NDV for direct administration to cancer patients. In the first documented human use of NDV, administration of the mesogenic NDV Hickman strain to a patient with acute myelogenous leukemia resulted in reduction in leukemic blast count and transient improvement in symptoms [6]. In a case report, mesogenic NDV 73-T strain was used for intratumoral treatment of a patient with advanced cervical cancer, resulting in partial response [4]. Csatary and colleagues reported a case series of patients with various advanced cancers treated with mesogenic NDV strain MTH-68 using various routes of administration, with reported partial or even complete responses across a number of cancers [102,110]. In an additional series, fourteen patients with glioblastoma were treated intravenously with NDV MTH-68 on various schedules. Seven of the patients achieved response to therapy with four of the patients surviving between 5 and 9 years at the time of the publication in 2004 [111].

In the early 2000s, NDV strain PV701, derived from the mesogenic strain 73-T, was evaluated in three phase I trials in patients with advanced malignancies using intravenous administration [80,81,112,113]. In the initial study, in 79 patients there were two responses (one complete and one partial), with seven additional minor responses noted. In fourteen patients, a prolonged progression free survival that lasted from 4 to over 30 months was observed [112]. In a subsequent study of eighteen patients with various advanced cancers using slower infusion rate but higher therapeutic dosing, a higher response rate was observed, with demonstration of four major and two minor responses, with six patients surviving at least 2 years [80,81]. Despite the initial promising results, PV701 unfortunately was not evaluated in further studies, likely secondary to changes in regulatory guidelines surrounding the use of mesogenic and velogenic NDV strains. NDV strains that are highly virulent in birds are classified as USDA select agents, limiting their clinical applicability. Lentogenic NDV strain HUJ has been evaluated using an intravenous approach in 14 patients with recurrent glioblastoma, demonstrating a complete response in one patient. Across the studies, intravenous administration of NDV has in general been well tolerated, with flu-like symptoms being the most common reported adverse event.

While previous studies in humans have only explored naturally occurring NDV strains, genetically modified NDVs have recently entered therapeutic testing. As described above, recombinant NDV expressing GM-CSF (MEDI5395), also based on the 73-T strain, is being evaluated in patients with various advanced malignancies in combination with durvalumab using intravenous administration (NCT03889275). Additional recombinant NDVs are in various stages of development and are expected to enter clinic within the next year.

## 6. Conclusions

Over the past 60 years, NDV has repeatedly demonstrated its therapeutic potential, both as an oncolytic agent and an immunotherapeutic agent. With intravenous administration, NDV is one of the few viruses that has demonstrated an ability to result in partial and even complete responses as a single agent. Durability of these responses further highlights that the therapeutic effect of the virus is likely not solely dependent on direct oncolysis, but rather on the ability of the virus to induce durable immunity. While the use of mesogenic and velogenic (and thus most lytic) strains for antitumor therapy is limited due to their pathogenic potential in birds, data with fewer lytic strains nevertheless highlights their potential to incite antitumor immunity, with the recent data indicating their ability to potentiate the efficacy of systemic immune checkpoint inhibitors. Furthermore, with the advent of genetic engineering, it has become possible to modify NDV to further enhance its immunogenic potential, with introduction of transgenes targeting both innate and adaptive immune pathways. As with other oncolytic viruses, many questions surrounding therapy with NDV remain unanswered, including optimal route of administration, ideal strategies for genetic engineering, therapeutic sequencing with immune checkpoint inhibitors, and best combination partners. While preclinical syngeneic models have provided some answers to these questions, most, if not all, models fail to capture the heterogeneity of human cancers and are thus not sufficient for guiding therapy. It is thus imperative that within the context of clinical trials we collect as much information as possible, with translational endpoints being prioritized as essential elements of any study. Understanding of the evolution of immune response to the virus and the tumor, even in a trial with no clinical benefit, should be a key priority for any clinical trial utilizing oncolytic viruses, as it is the only way to guide the further development of these agents and move the field forward.
